# Mitochondrial unfolded protein response in regulatory T cell function: a protective mechanism in immune aging

**DOI:** 10.3389/fimmu.2025.1621759

**Published:** 2025-06-30

**Authors:** Lillie Lewis, Deepa Valvi, Roberto Gedaly, Francesc Marti

**Affiliations:** ^1^ Department of Surgery - Transplant Division, College of Medicine, University of Kentucky, Lexington, KY, United States; ^2^ Lucillle Parker Markey Cancer Center, College of Medicine, University of Kentucky, Lexington, KY, United States; ^3^ Alliance Research Initiative (TILT Alliance), College of Medicine, University of Kentucky, Lexington, KY, United States

**Keywords:** regulatory T-cells, cell metabolism, cellular stress, oxidative stress, unfolded protein response, immunosenescence, aging

## Abstract

Age-related conditions, such as neurodegenerative disease, cancer, and autoimmune disorders, are increasingly recognized as closely linked with the gradual deterioration of the immune system. Regulatory T cells (Tregs) are a small, specialized subset of T lymphocytes that play a critical role in maintaining immune homeostasis and self-tolerance. As individuals age, Treg cells demonstrate reduced capacity to suppress some autoreactive immune responses, although they largely retain their capacity to regulate effector antiviral and antitumor immunity. Unlike conventional effector T cells (Teff), which primarily derive energy from glycolysis, Tregs rely more on mitochondrial oxidative phosphorylation to fulfill their energy requirements. This metabolic profile renders them particularly sensitive to mitochondrial dysfunction, underpinning the critical role of mitochondrial protective pathways in preserving the functional integrity of Treg cells. The mitochondrial unfolded protein response (mitoUPR) is gaining special relevance among these protective mechanisms. In this review, we examine the complex interplay between immune aging and mitochondrial dynamics, with particular emphasis on the essential role of mitoUPR in supporting Treg function. We further discuss how targeting mitochondrial stress responses may offer novel therapeutic avenues for age-related diseases characterized by Treg dysfunction.

## Introduction

Aging is characterized by a progressive decline in physiological processes across the lifespan of an organism, with the immune system among the most profoundly affected (1). This age-associated immune deterioration, a phenomenon known as *immunosenescence*, contributes to the higher susceptibility to infections, chronic inflammation, and age-related diseases (2). Notably, different immune cell types exhibit distinct responses to aging, likely reflecting their different metabolic demands and stress response mechanisms.

Regulatory T cells (Tregs) have emerged as a key population in the context of immunosenescence (3–6). This small subset of T cells plays a critical role in maintaining immune homeostasis by promoting tolerance and preventing excessive inflammatory responses in both innate and adaptive immune arms (7, 8). Treg cells possess a distinct metabolic profile. Unlike conventional effector T (Teff) cells, Tregs rely heavily on mitochondrial fatty acid oxidation (FAO) and oxidative phosphorylation (OXPHOS) to sustain their suppressive function (9). As such, Treg cells are particularly vulnerable to agents that compromise mitochondrial function, including reactive oxygen species (ROS), environmental toxins, and genotoxic stressors, which tend to accumulate with age (1, 10, 11). To preserve their functional integrity, Tregs engage different mitochondrial protective mechanisms, including antioxidant pathways, DNA repair mechanisms, and mitochondrial quality control systems (12–16).

One of such mitochondrial quality control systems is the mitochondrial unfolded protein response (mitoUPR). The mitoUPR responds to the accumulation of unfolded or misfolded mitochondrial proteins by upregulating the transcription of chaperones and proteases (17, 18). Disruption of mitoUPR results in the accumulation of dysfunctional mitochondria or misfolded mitochondrial proteins, which may contribute to the onset and progression of a variety of age-related pathologies such as neurodegenerative disorders, metabolic syndromes, and systemic inflammation (19, 20). We have recently demonstrated that Tregs exhibit increased baseline expression of different mitoUPR proteins compared to conventional T cells (Tconv) (21).

This review explores the dynamic interplay between immune metabolism and aging, with a particular focus on Treg cells and the relevance of the mitoUPR in sustaining Treg function and driving their fate.

## Immune cells in aging

A properly functioning immune system is essential for health and disease protection. With advancing age, immunosenescence leads to increased vulnerability to chronic inflammation, infectious diseases, and reduced vaccine responsiveness in the elderly population (22).

The innate immune system serves as the body’s first line of defense against infections and injuries, providing a rapid and non-specific response already active from birth (23–25). Key components of the innate immune system include physical barriers such as the skin and mucosal membranes, along with chemical and cellular defenses (23). Most innate immune cells, except for natural killer (NK) cells, derive from myeloid progenitors in the bone marrow (26). Dendritic cells (DCs) and NK cells bridge the innate and adaptive responses by alerting and activating T cells (27, 28). Meanwhile, other innate immune cells neutralize pathogens by releasing cytotoxic chemicals, cytokines, chemokines, and antimicrobial peptides, or by direct pathogen elimination via phagocytosis (29). Traditionally viewed as lacking memory, the innate immune system is now recognized to exhibit trained immunity – a long-term functional reprogramming, leading to an enhanced responsiveness to subsequent, even unrelated, challenge (30). This response is not antigen-specific but rather represents a broad, heightened state of readiness in the cellular innate immune system (30–32).

In aging individuals, innate immune cells exhibit many types of functional impairment, hindering their ability to facilitate tissue repair and properly initiate adaptive responses (33). DCs exhibit impaired antigen uptake and dysfunctional T cell priming, sometimes leading to the activation of T cells in response to self-DNA (34, 35). NK cells in aged individuals secrete less interferon gamma (IFNγ), a key cytokine for T cell differentiation (36, 37). Neutrophils exhibit reduced migration and impaired production of neutrophil extracellular traps (NETs), essential for microbial neutralization and elimination (38–40). Macrophages display impaired chemotaxis and increased ROS production, compromising their ability to clear pathogens (41). Additionally, in the elderly, microglial cells in the central nervous system become sensitized by the chronic, low-grade inflammation that develops with aging, a condition known as inflammaging (42–44).

The adaptive immune system, while slower to respond than the innate immune system, is characterized by its antigen specific response and the generation of immunological memory (25). Activation of adaptive immunity is initiated through the recognition of individual antigens by clonotypic, highly specific receptors expressed on B and T lymphocytes, enabling targeted and long-lasting immune responses (45, 46). In the bone marrow, common lymphoid progenitors develop from hematopoietic stem cells (26). These progenitors give rise to committed T cell, B cell, or NK cell precursors. Once committed to the T cell lineage, T cell precursors migrate to the thymus where they become CD4^+^ helper T cells, CD8^+^ cytotoxic T cells, or Treg cells (47). After their thymic release, following antigen exposure, CD4^+^ and CD8^+^ T cells differentiate into memory T cells that exhibit enhanced responsiveness upon re-exposure to the same antigen (48). In adulthood, thymic involution leads to a progressive decline in the output of naïve T cells (49); however, the peripheral T cell pool is maintained through homeostatic proliferation and survival within secondary lymphoid tissues (50, 51). This shift from thymic output to peripheral T cell generation dynamics imposes a replicative stress, thereby increasing the risk of replication-induced genomic instability and cellular senescence, and ultimately contributing to functional decline and eventual exhaustion of the T cell population (52, 53).

**Figure 1:**
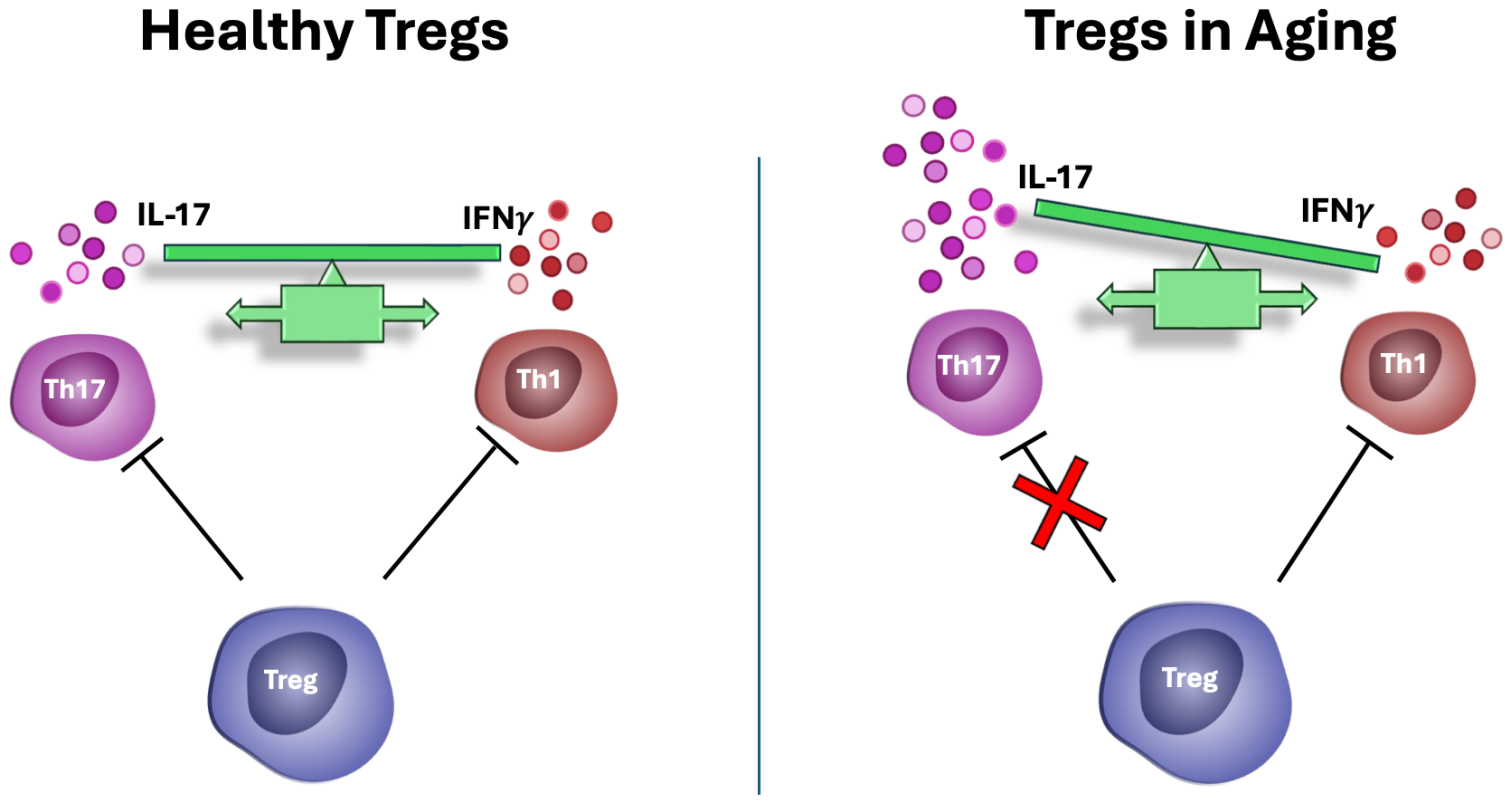
Tregs in aging. In aged individuals, Tregs lose their capacity to suppress IL-17 mediated autoimmunity but retain their ability to suppress antigen presentation, as well as antiviral, and antitumor immune responses. This imbalance may contribute to the simultaneous increased risk for autoimmunity and cancer with aging.

Unlike other T cell subsets, the Treg cell compartment remains stable or even expands with age (54–56). While aged Tregs retain many of their core suppressive functions, some regulatory mechanisms become selectively impaired (**Figure 1**). Notably, their ability to control the proliferation of IL-17 producing T helper cells (Th17) is compromised in chronic, but not acute, inflammatory conditions (57, 58). In contrast, aged Tregs continue to effectively suppress antigen-presenting cell function and IFN-γ production (58), which is critical for antiviral and antitumor immunity and promoting the expression of immune checkpoint inhibitors (59). In addition, Tregs in aged mice are less efficient in suppressing IL-2 production of effector T cells (60). This selective dysregulation in Treg function during aging may contribute to the paradoxical combination of weakened immunity against infections and increased autoimmune responses observed in the elderly, underscoring the importance of Treg cells in aging-related immune remodeling and their relevance as targets for immunotherapeutic strategies in aging. The distinct metabolic profiles of glycolytic-driven Teff and mitochondrial-reliant Treg cells (9) has led to extensive investigation into the role of mitochondrial function in maintaining the functional integrity of Tregs.

## Mitochondrial function in Tregs

**Figure 2:**
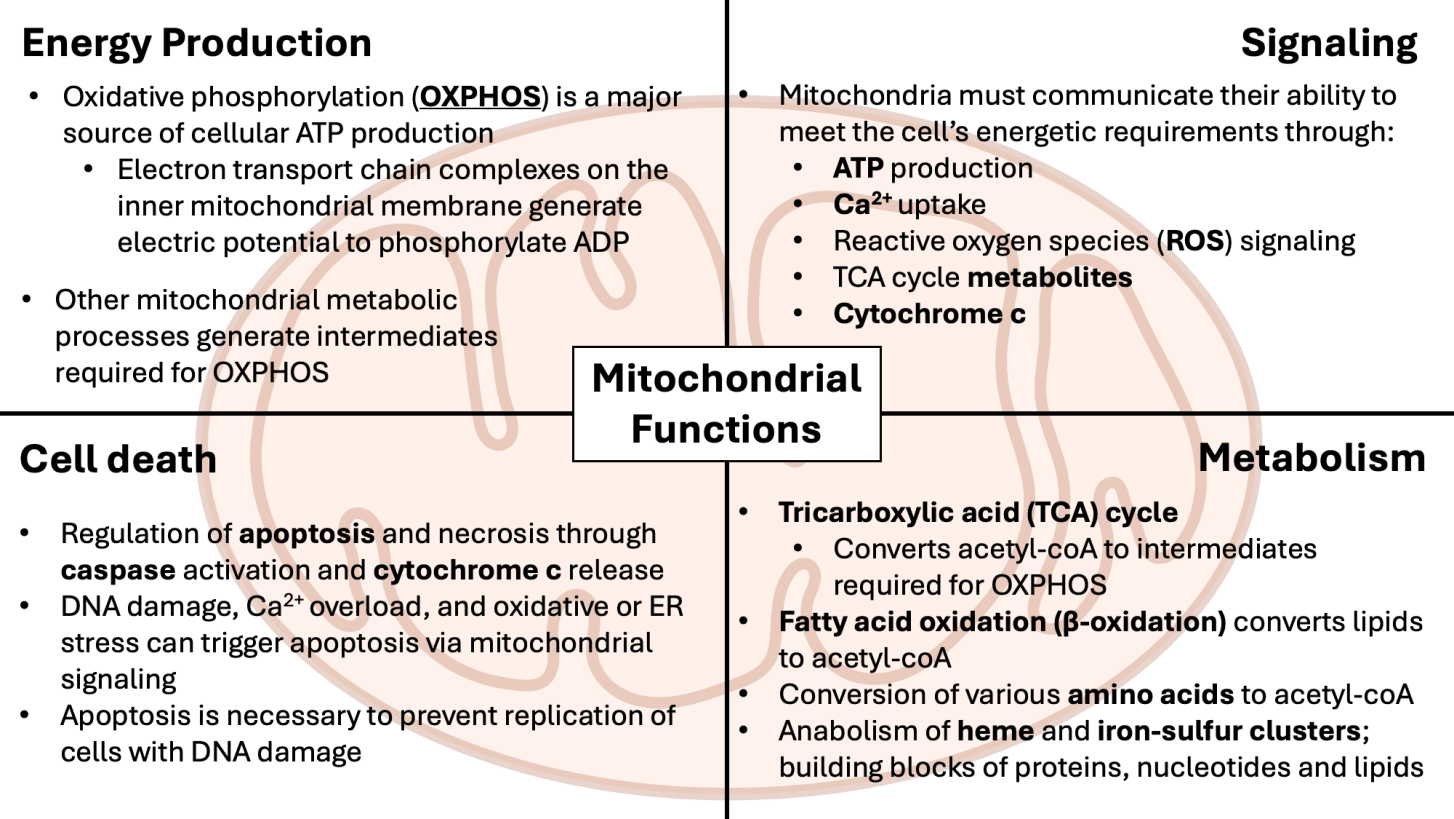
Overview of mitochondrial functions. Mitochondria are membrane-bound metabolic and signaling organelles with their own circular DNA. Within the matrix, separated by the inner mitochondrial membrane, ATP is produced from the tricarboxylic acid (TCA) cycle and the electron transport chain (ETC). β-oxidation contributes to ATP production by breaking down fatty acids into acetyl-CoA required for the TCA cycle. Production of ATP from mitochondria regulates nutrient-sensing via AMPK/mTOR signaling, while mitochondrial reactive oxygen species (mtROS) from the ETC, at low levels, activates the ERK signaling cascade, promoting proliferation and cell growth.

Mitochondria are intracellular, membrane-bound organelles with diverse roles in cellular metabolism and homeostasis (61), as outlined in **Figure 2**. In the immune system, mitochondria are essential for regulating inflammation and determining cell development, fate and function (62). Mitochondria drive inflammatory signaling through the release of mitochondrial damage-associated molecular patterns (mtDAMPs), including mitochondrial ROS (mtROS), mitochondrial N-formyl peptides, and mtDNA (63). These mtDAMPs act as endogenous “alarm signals” that are recognized by pattern recognition receptors (PRRs) such as RIG-1-like receptors (RLRs), NOD-like receptors (NLRs), and Toll-like receptors (TLRs) expressed predominantly in cells of the innate immune system (64). Engagement of PRRs triggers downstream signaling cascades that lead to the induction of pro-inflammatory cytokines and tissue-repair intermediates (65). In addition to playing a major role in initiating immune responses to both microbial pathogens and sterile insults caused by cell death or tissue damage, mitochondrial signaling and metabolism regulate the differentiation and functional programming of immune cells (62, 66–68). Mitochondrial activity also influences both the production of cytokines and the responsiveness of immune cells to cytokine-mediated signals (69, 70). The importance of mitochondrial health in immune regulation is further highlighted by the observation that many immunosenescence-associated alterations are closely linked to progressive mitochondrial dysfunction (71).

In Treg cells, mitochondria play a unique role in their survival and function (9, 14, 72–75). Mitochondrial metabolism, especially FAO and OXPHOS are essential for the *in vitro* suppressive abilities of Tregs (9). In contrast, Tconv use glycolytic metabolism for rapid energy production to support their effector functions (9). Genetic studies further underscore the importance of mitochondrial integrity in maintaining the suppressive function of Treg cells, as the deletion of the mitoUPR-related proteins SIRT3 or PGC1-α leads to impaired Treg suppressive activity (72). Different lines of evidence also support a critical role of mitochondrial metabolism in the differentiation of Tregs from naïve T cells (14, 76). Interestingly, in the tumor microenvironment, disruption of OXPHOS and lipid metabolism in tumor-infiltrating Tregs has been shown to paradoxically bolster their suppressive capacity, mediated in part by type I IFN signaling and increased IL-10 production (77), also demonstrating that not all Treg suppressive functions are dependent on lipid metabolism and OXPHOS. Likewise, freshly isolated *ex vivo* human Tregs exhibit high glycolytic activity (78). However, disruptions in mitochondrial OXPHOS can increase oxidative stress, potentially compromising Treg survival by inducing apoptosis (79). Although excessive oxidative stress is detrimental to Treg cell function, low levels of ROS are essential for intracellular signaling and proper immune regulation (80). mtROS, particularly hydrogen peroxide (H_2_O_2_), is an important second messenger that regulates key transcriptional pathways, including nuclear factor kappa B (NFκB) signaling essential for Treg development (80, 81), as well as nuclear factor of activated T cells (NFAT) and activator protein 1 (AP-1), which are involved in T cell signaling and gene expression (82). Additionally, mtROS play pivotal roles in Treg induction by macrophages and in the suppressive function of thymus-derived Tregs (83, 84). These findings illustrate the complex role of ROS in Treg biology, acting as both essential signaling molecules and potential stressors, depending on their levels and cellular context.

Mitochondrial dynamics of fusion and fission are essential for maintaining mitochondrial integrity and function under dynamic cellular conditions (85), and play a pivotal role in Treg cell biology. Mitochondrial fusion is a protective mechanism that mitigates cellular stress by enabling the exchange of mitochondrial DNA, proteins, and metabolites between damaged and healthy mitochondria (86). This process is largely mediated by the GTPases mitofusin 1 (MFN1), mitofusin 2 (MFN2), and optic atrophy protein 1 (OPA1) (85). Mitochondrial fusion is a crucial metabolic checkpoint necessary to promote Treg differentiation, lineage commitment and enhancing their suppressive function (87, 88). Conversely, mitochondrial fission – the division of mitochondria into smaller units – is mediated primarily by dynamin-related protein 1 (DRP1) (89). While mitochondrial fission is not required for Treg differentiation (85), its inhibition increases Treg accumulation in the central nervous system and spleen in animal models of autoimmune disease, such as multiple sclerosis (90). Mitochondrial fission remains essential for maintaining the health of the mitochondrial network (91). When fusion alone is insufficient to ameliorate damage from dysfunctional mitochondrial fragments, fission facilitates the segregation of these fragments, which can then be targeted for degradation through mitophagy, a selective form of autophagy specific to mitochondria (92). Defective mitophagy has been implicated in Treg dysfunction in autoimmune diseases such as myasthenia gravis, underscoring the critical role of mitochondrial quality control in preserving Treg function and immune homeostasis (13, 93).

## Mitochondrial dysfunction in aging

**Figure 3:**
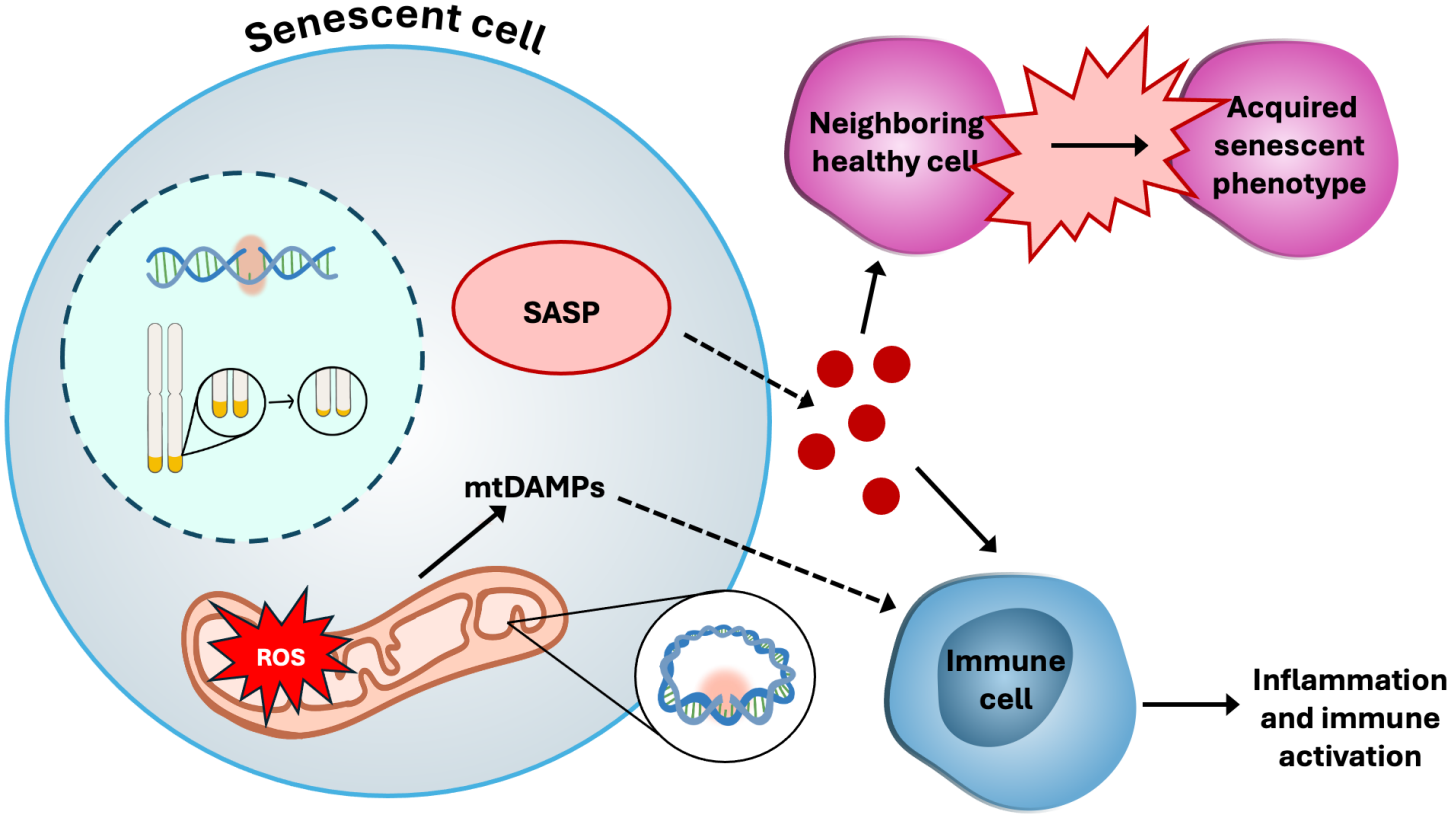
Cellular senescence. Senescent cells display many dysfunctional characteristics, such as: hyper-fused and elongated mitochondria; increased production of ROS, driving genomic instability in mitochondrial DNA (mtDNA) as well as nuclear DNA; release of mitochondrial damage-associated molecular patterns (mtDAMPs) such as mtROS and mtDNA, which stimulate inflammatory responses from immune cells; telomere shortening; and the senescence-associated secretory phenotype (SASP), which includes growth factors and pro-inflammatory signaling molecules.

Given the unique and critical role of mitochondria in Treg cell proliferation, metabolism and suppressive function, mitochondrial dysfunction poses a significant challenge – particularly in aging populations – by simultaneously driving inflammatory signaling and impairing Treg cell function (19, 93). This dual threat contributes to the age-associated decline in immune homeostasis and the increased prevalence of chronic inflammation. A major source of mitochondrial damage in the elderly is the accumulation of oxidative damage from mtROS generated as byproducts of OXPHOS (94). During OXPHOS, electron leakage from the electron transport chain (ETC) produces highly reactive superoxide anion radicals (O_2_^* −^), which are capable of degrading lipids, proteins, and nucleic acids within the cell (95). The enzyme superoxide dismutase (SOD) serves as a key mitochondrial antioxidant defense, converting O_2_^* −^ into H_2_O_2_, a less reactive but still potent signaling molecule (95). H_2_O_2_ is essential for the regulation cell cycle signaling by modifying redox-sensitive amino acids such as methionine and cysteine on target proteins (96). However, excessive accumulation of H_2_O_2_ can lead to oxidative stress and cellular damage (97). To prevent this, cells rely on a tightly regulated antioxidant system comprising catalase (CAT), glutathione peroxidase (GPx), and peroxiredoxins (Prx) (98, 99), which detoxify H_2_O_2_ and maintain redox balance. The relationship between ROS production and mitochondrial function is complex and context dependent. Different studies have reported conflicting correlations between ATP production and ROS levels (100, 101), as well as between ROS production and mitochondrial membrane potential (ΔΨm). These discrepancies likely reflect variations in experimental models, cell types, and physiological conditions, highlighting the nuanced interplay between mitochondrial bioenergetics and redox signaling. Under normal physiological conditions, ATP production, ΔΨm, and ROS levels are positively correlated because the cell can efficiently neutralize ROS. However, under stress conditions, the cell struggles to neutralize ROS, resulting in damaged OXPHOS machinery and a decline in both ΔΨm and ATP production. In conditions like type 2 diabetes and obesity, when ΔΨm is impaired, there is a disruption of the electrochemical gradient necessary for the import of substrates like pyruvate and fatty acids (102–104). As a result, these substrates, along with proteins that fail to be imported, accumulate in the cytosol and activate the mitoUPR) (18, 105). The cytosolic accumulation of ROS and misfolded proteins due to mitochondrial stress leads to various forms of cellular dysfunction, including cellular senescence (**Figure 3**), which is characterized by the cessation of proliferation and resistance to cell death and apoptosis (106). In addition to mitochondrial dysfunction, other stressors can contribute to cellular senescence, including oncogenic stress, telomere attrition, replicative stress, and irradiation – all of which accumulate with age and create a cycle of increasing toxicity within the intracellular environment of the senescent cell (107). A key hallmark of mitochondrial dysfunction and cellular senescence is genomic instability, which contributes to the accumulation of DNA mutations and is associated with an increased risk of malignancy (10). Telomere shortening, a defining feature of both aging and cellular senescence, is also closely linked to mitochondrial dysfunction (1, 10, 108). Although the precise causal relationship between telomere attrition and mitochondrial impairment remains unclear, growing evidence suggests the bidirectional crosstalk between these two processes, wherein telomere damage can influence mitochondrial function and *vice versa* (10, 108–110). The deleterious effects of senescence are not confined to the senescent cells themselves. Senescent cells actively secrete a range of pro-inflammatory cytokines, chemokines, growth factors, and proteases that can induce secondary senescence in surrounding healthy cells and tissues – a phenomenon known as the senescence-associated secretory phenotype (SASP) (111, 112), which can contribute to amplify the chronic inflammation that drives aging and its associated pathologies.

Given the complex, self-reinforcing interplay among mitochondrial dysfunction, cellular senescence and inflammatory responses, the regulatory role of Treg cells in controlling these inflammatory loops is critical for maintaining immune and tissue homeostasis. In centenarians, Tregs maintain a strongly anti-inflammatory secretory profile, accompanied by reduced levels of pro-inflammatory cytokines compared to those observed in the general aging population (4). This observation suggests a potential role for Tregs in mitigating inflammaging. However, recent studies have shown evidence that senescent Tregs can accumulate with age and may, paradoxically, contribute to the progression of inflammaging and age-related pathologies (3, 5, 113). Because Tregs are particularly vulnerable to mitochondrial dysfunction, they may be more susceptible to mitochondria-driven senescence than Tconv, as demonstrated in mice (5).

Chronic inflammation in elderly populations, often driven by mitochondrial dysfunction, is a key contributor to the process of inflammaging. In this context, Treg activity is essential for suppressing the excessive inflammatory responses and managing the low-grade, subclinical inflammation associated with aging. This underscores the great therapeutic potential of targeting mitochondrial pathways to enhance Treg function and mitigate inflammatory signaling. Among these pathways, the mitoUPR stands out as a particularly promising target, given its central role in preserving mitochondrial integrity and cellular homeostasis under stress conditions.

## mitoUPR in Treg cells

### Activation of the mitoUPR

The UPR was initially identified in the endoplasmic reticulum (ER) as a protective mechanism activated by ER stress (ERS) (114). ERS occurs upon accumulation of unfolded or misfolded proteins in the ER due to homeostatic disruptions such as oxidative stress or Ca^2+^ imbalance. More recently, a similar pathway -the mitochondrial UPR (mitoUPR)- has been identified in mitochondria, where stress or damage initiates the transcriptional activation of genes encoding molecular chaperones and proteases to restore mitochondrial function (18, 115).

**Figure 4:**
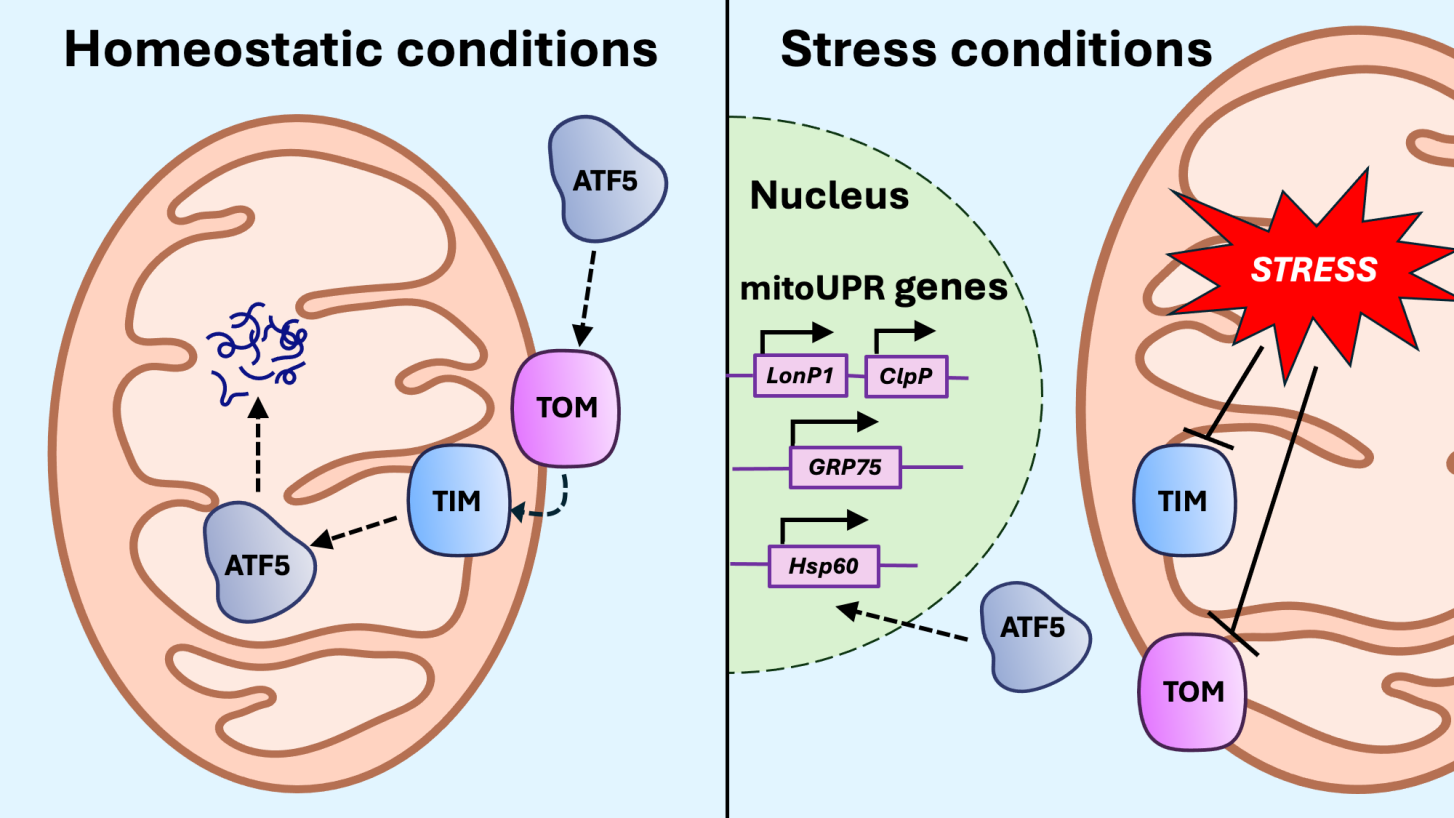
Activation of the mitoUPR. Under conditions of mitochondrial stress, protein import to the mitochondria through the translocase of the outer membrane (TOM) and translocase of the inner membrane (TIM) is impaired, preventing the import of activating transcription factor 5 (ATF5). When ATF5 cannot be imported into the mitochondria, it translocates to the nucleus, where it upregulates transcription of mitoUPR genes, including proteases LonP1 and ClpP, as well as chaperones GRP75 and Hsp60.

Any process that compromises mitochondrial protein translation, synthesis, proteostasis, import or degradation can trigger the mitoUPR (116–120). Among these triggers are the mtROS accumulation, the development of the mitochondrial permeability transition pore (mPTP), mito-nuclear protein imbalance, reduced ΔΨm, the overactivation or depletion of mitochondrial chaperones and proteases, and the depletion of mitochondrial prohibitins (121–124). One key activating signal for the mitoUPR is the activating transcription factor associated with stress 1 (ATFS-1) in *C. elegans* and its mammalian homolog, activating transcription factor 5 (ATF5) (115, 125). When the mitochondrial protein import machinery is functional, ATF5 is efficiently translocated into the mitochondria and subsequently degraded. In contrast, when protein import is compromised, ATF5 is redirected to the nucleus where it activates the transcription of mitoUPR genes (**Figure 4**), including chaperones and proteases such as heat shock proteins (HSPs) like mitochondrial Hsp70 (mtHsp70 or GRP75) and Hsp60, Lon protease (LonP1), and caseinolytic mitochondrial matrix peptidase proteolytic subunit (ClpP) (125, 126). Chromatin remodeling also regulates the nuclear transcription of mitoUPR genes (18). Under mitochondrial stress, chromatin is compacted by histone methyltransferases, but specific DNA regions encoding mitoUPR genes are protected from compaction by demethylases (115). Notably, disruptions in Ca^2+^ homeostasis and mitophagy by gene knockdown do not trigger the mitoUPR, suggesting that both processes are required for mitoUPR activation (116, 127, 128).

Activation of the mitoUPR in mammals is closely linked to the integrated stress response (ISR), a conserved pathway activated by a variety of cellular stressors (129). ISR is initiated by a set of eukaryotic initiation factor 2 alpha (eIF2α) kinases, including protein kinase R-like endoplasmic reticulum kinase (PERK), protein kinase R (PKR), heme-regulated inhibitor (HRI), and general control nonderepressible 2 (GCN2) (130). These kinases phosphorylate eIF2α, promoting the translation of stress-responsive transcription factors such as ATF4, CHOP, and ATF5, which in turn upregulate genes encoding mitochondrial chaperones and proteases involved in the mitoUPR (115, 131). During ISR activation, stress granules (SG) facilitate the nuclear relocation of ATF5 (132). However, while ISR-related transcription factors are essential for mitoUPR activation, the ISR alone is not sufficient to fully activate the mitoUPR (122) and additional mitochondrial-specific signals are required to initiate a complete stress response. One of these additional signals is mtROS. mtROS contribute to the mitoUPR coordination by oxidizing heat shock protein 40 (HSP40). In this oxidized form, HSP40 interacts with mitochondrial preproteins in the cytosol before being imported into the mitochondria, where it associates with HSP70. The accumulation of HSP40-HSP70 complexes with mitochondrial protein precursors promotes the nuclear translocation of the transcription factor heat shock factor 1 (HSF1), where it upregulates the transcription of mitoUPR genes. Although HSF1 has long been recognized as a central regulator of cellular stress responses, its specific role in mitoUPR activation has only recently been identified (133–135).

Interestingly, and similar to the non-cell autonomous nature of the senescence-related SASP phenomenon, mitoUPR activation can extend beyond the stressed cell in neighboring cells, suggesting a form of paracrine mitochondrial communication. In *C. elegans*, mitochondrial stress in neurons not only activates mitoUPR within the neurons, but also in distal intestinal cells (136–139) by the secretion of serotonin and metabolic signaling molecules from stressed neuronal mitochondria (137, 140, 141). Whether this paracrine mitoUPR signaling occurs in other cellular systems is not yet known.

### mitoUPR signaling

Research over the last decade has revealed that mitoUPR operates as a multi-axis system in coordination with other cellular processes and stress responses. This multi-axis regulation includes the canonical axis, the sirtuin axis, the intermembrane space/estrogen receptor alpha (ERα) axis, and the translation axis (129). These different axes work together to preserve mitochondrial function in response to both cellular and mitochondrial stress.

**Figure 5:**
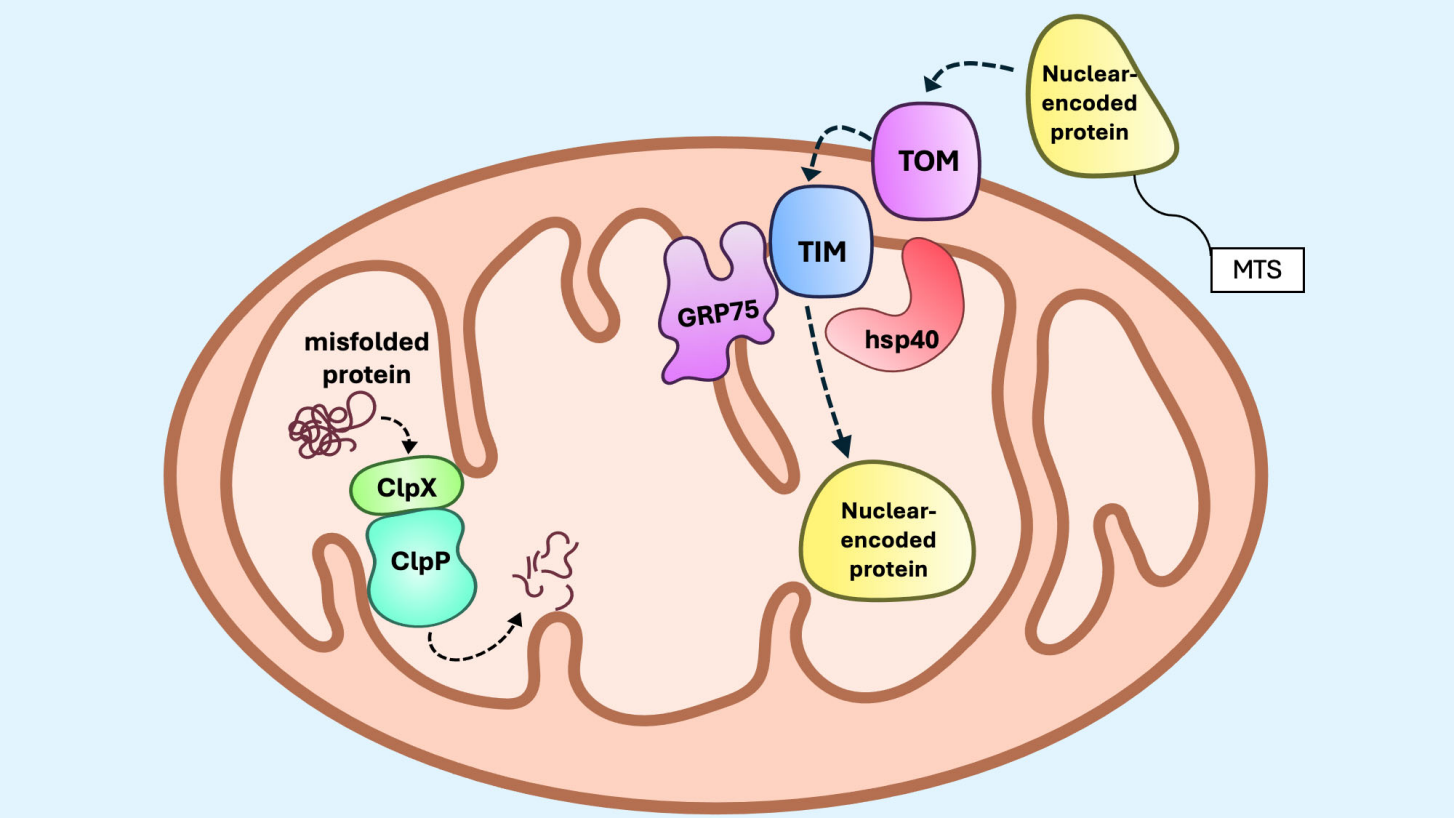
The mitoUPR canonical axis. The canonical mitoUPR consists of chaperones and proteases that control protein folding and degradation within mitochondria. GRP75, along with Hsp40, assist with protein folding during import of nuclear-encoded proteins with mitochondrial targeting sequences (MTS) to the matrix. When misfolded proteins or protein aggregates accumulate, ClpP and ClpX form a protease complex that degrades misfolded proteins. During proteotoxic stress, the expression of these chaperones and proteases are upregulated within mitochondria.

The canonical axis of the mitoUPR is defined by the upregulation of mitochondrial molecular chaperones and proteases that function to disassemble, refold, or degrade misfolded or aggregated proteins (**Figure 5**). The two main protein-folding systems that operate in the mitochondrial matrix are the chaperone glucose-regulated protein 75 (GRP75, also known as mtHsp70) and the chaperonin Hsp60 (142–144). GRP75 plays a central role during the import and folding of proteins translocated into the matrix, whereas Hsp60 functions primarily to refold misfolded or partially unfolded proteins within the matrix. GRP75 associates with multiple protein complexes along the inner mitochondrial membrane (IMM), contributing not only to protein assembly, folding and import, but also to the regulation of mitoUPR signaling (145). GRP75 cooperates Hsp40 (DNAJA3) to create an efficient ATP-dependent chaperone system that ensures the fidelity of mitochondrial protein folding and assembly (144). Proteolytic degradation of irreversibly damaged proteins during mitoUPR is mediated by protease complexes such as ClpXP. The ClpX subunit recognizes and binds to specific degradation motifs on substrate proteins, partially unfolding them for translocation into the ClpP protease chamber, where proteolysis occurs (146). Overexpression of ClpX, but not ClpP, during myogenesis, increases de expression of mitoUPR components and enhances OXPHOS activity via induction of the transcription factor C/EBP homologous protein (CHOP) (147). This finding suggests that ClpX may exert regulatory control over mitoUPR signaling and mitochondrial metabolism independent of its proteolytic partner. Emerging evidence also indicates that the mitoUPR intersects with broader cellular developmental programs. Components of the canonical mitoUPR have been implicated in the regulation of follicular cell development, supporting a model in which mitochondrial proteostasis pathways are integrated into signaling networks that govern cellular differentiation and function (148, 149). These insights expand the functional scope of the mitoUPR beyond stress adaptation, positioning it as a key regulator of both mitochondrial and cellular homeostasis.

**Figure 6:**
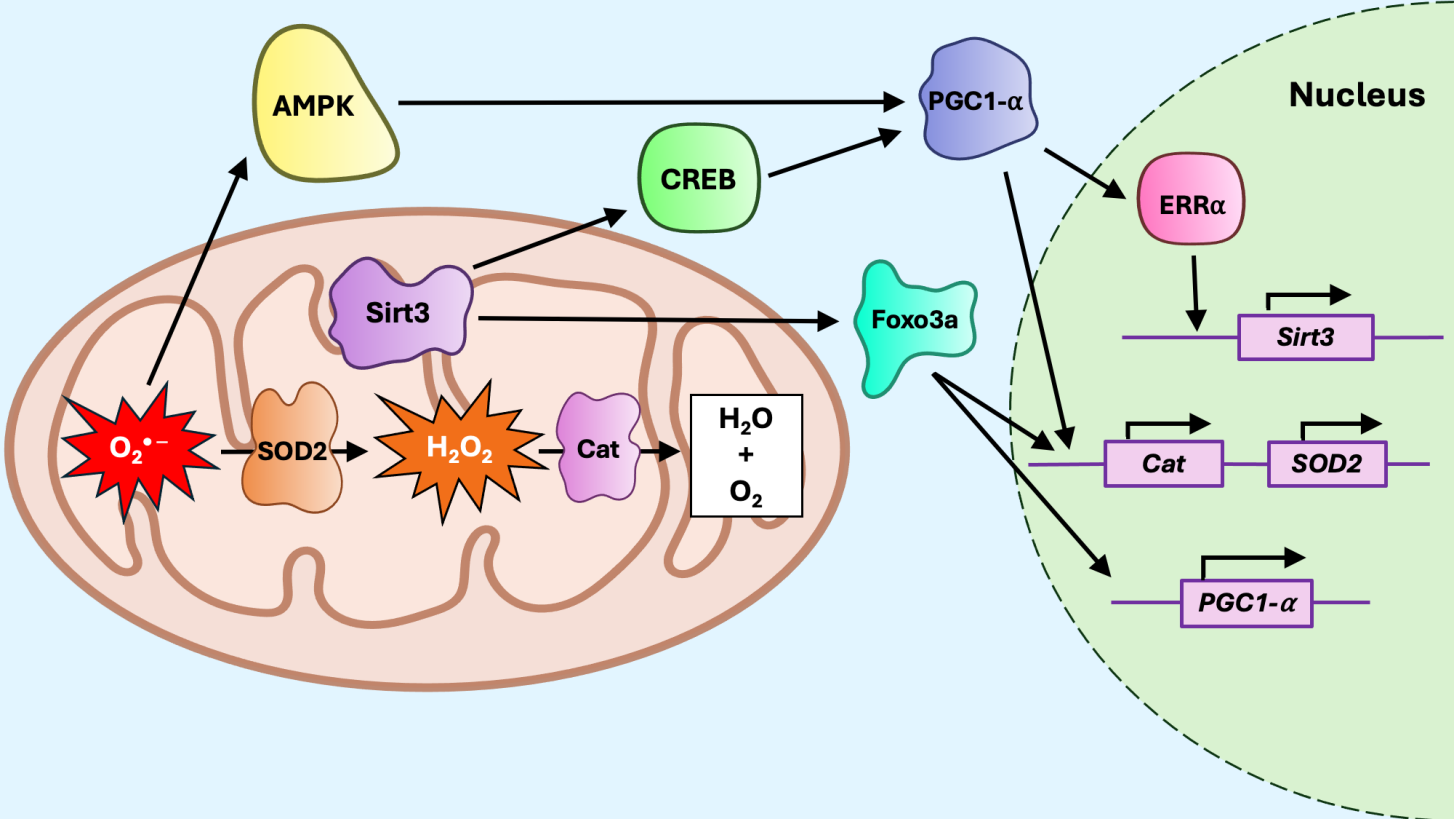
The mitoUPR sirtuin axis. Reactive oxygen species, including superoxide anion (O_2_^* −)^ and hydrogen peroxide (H_2_O_2_) promote activation of Sirt3. Sirt3 deacetylates CREB, which activates PGC1a, and Foxo3a. Foxo3a promotes transcription of *PGC1a*, but also interacts with PGC1a to promote transcription of antioxidant genes such as *Catalase* (*Cat*) and *superoxide dismutase* (*SOD2*). PGC1a can also upregulate Sirt3 expression by activating estrogen-related receptor alpha (ERRa), which promotes *Sirt3* transcription. Reactive oxygen species are neutralized by upregulation of SOD2, which converts O_2_^* −^ to H_2_O_2_, and Cat, which converts H_2_O_2_ to water and oxygen.

The sirtuin axis (**Figure 6**) increases resistance to mtROS by upregulating antioxidants and coordinating the mitophagy response (150, 151). The NAD^+^-dependent deacetylases of the sirtuin family SIRT3 and SIRT1, have been identified as mitoUPR regulators (129). SIRT1 locates in the nucleus and cytosol, while SIRT3 is primarily found in the mitochondria and also, more recently, in the nucleus, though its nuclear function remains unclear (152, 153). mtROS stimulates AMP-activated protein kinase (AMPK), which activates peroxisome proliferator-activated receptor gamma coactivator 1 alpha (PGC-1α), leading to upregulation of SIRT3 via estrogen-related receptor alpha (ERRα) (154–156). Interestingly, the interplay between SIRT3 and PGC-1α is reciprocal, since SIRT3 may also promote PGC-1α activity through multiple mechanisms, including the activation of upstream regulators such as CREB (155, 157), or the deacetylation of forkhead box class O 3a (FOXO3a) (155, 158). PGC-1α and FOXO3a interact to upregulate antioxidants SOD2 and CAT, which neutralize mtROS in a rapid two-step reaction (159) whereby SOD2, along with the cofactor manganese, efficiently converts O_2_^* −^ into H_2_O_2_ and oxygen (160), and CAT rapidly converts H_2_O_2_ into water and oxygen (161, 162). The bidirectional SIRT3/PGC-1α regulation forms a positive feedback loop that amplifies the mitochondrial adaptive response under conditions of metabolic stress. Although not directly indicative of mitoUPR engagement, the activation of the SIRT3/PGC-1α/FOXO axis complements the canonical mitoUPR during proteotoxic stress, contributing to the overall mitochondrial stress response. Disruption of this pathway has been implicated in metabolic disorders, including those associated with aging (163–166).

**Figure 7:**
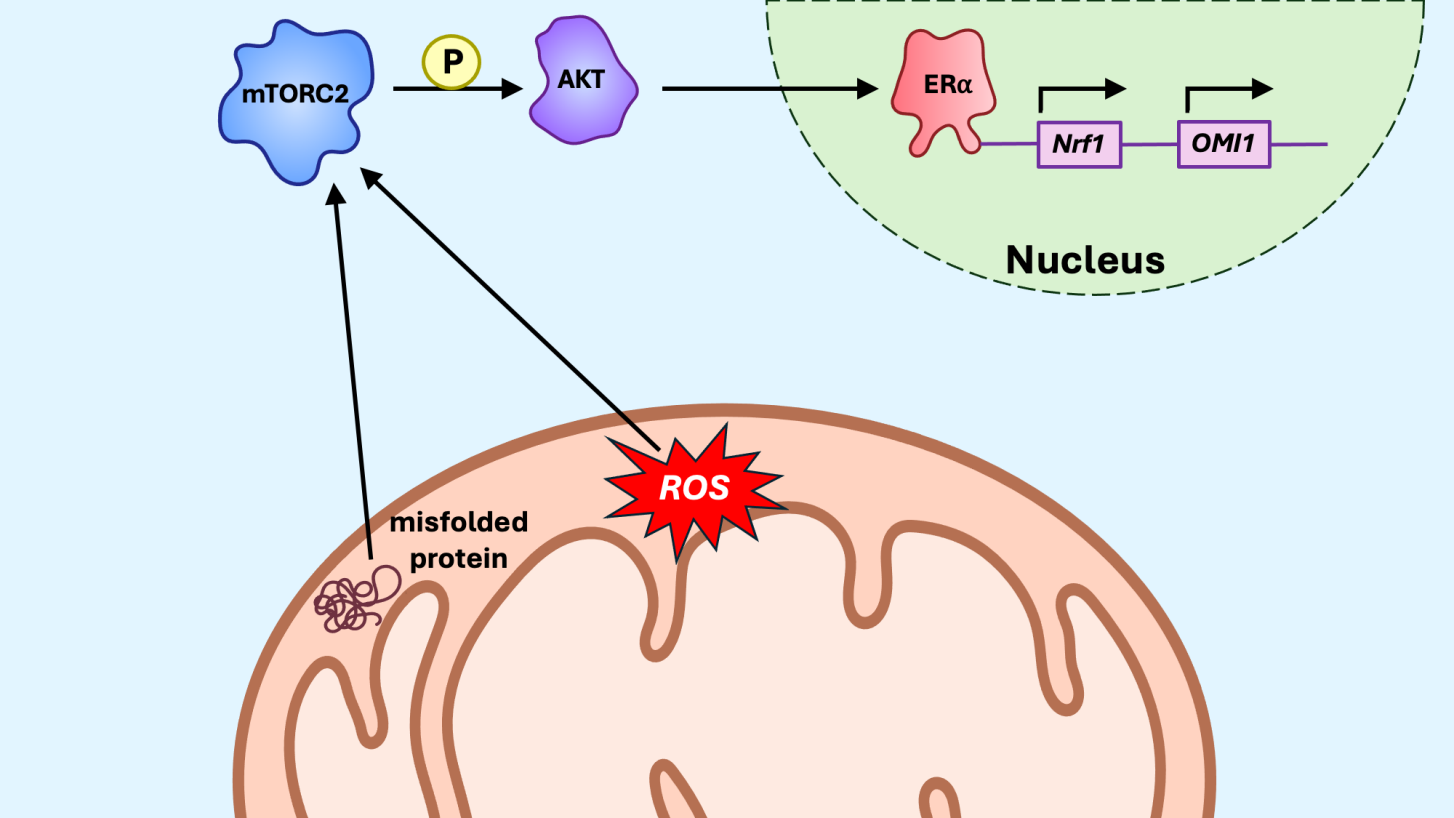
The mitoUPR ERα axis. In response to oxidative stress in the intermembrane space (IMS), estrogen receptor alpha (ERα), via mTORC2/AKT signaling, promotes transcription of the protease *OMI1*, which degrades misfolded proteins within the IMS. ERα also promotes transcription of *Nrf1*, a transcription factor promoting transcription of nuclear-encoded components of the electron transport chain (ETC).

The ERα axis of the mitoUPR (**Figure 7**) in the intermembrane space (IMS) activates estrogen receptor alpha (ERα) via AKT signaling and ROS production, leading to increased levels of nuclear respiratory factor 1 (Nrf1) and the protease OMI1 (167). Overall, ERα activation increases proteasome and protease expression levels, thereby regulating protein quality control within the IMS. Notably, small heat shock proteins (sHSPs) act as chaperones in the IMS, both under homeostatic conditions and in response to protein aggregation (168). In cells that do not express ERα, CHOP and Hsp60 are induced in response to IMS stress, suggesting that, although the ERα axis operates independently of the canonical mitoUPR, the canonical axis may compensate when the ERα axis is insufficient to neutralize proteotoxic stress (169).

**Figure 8:**
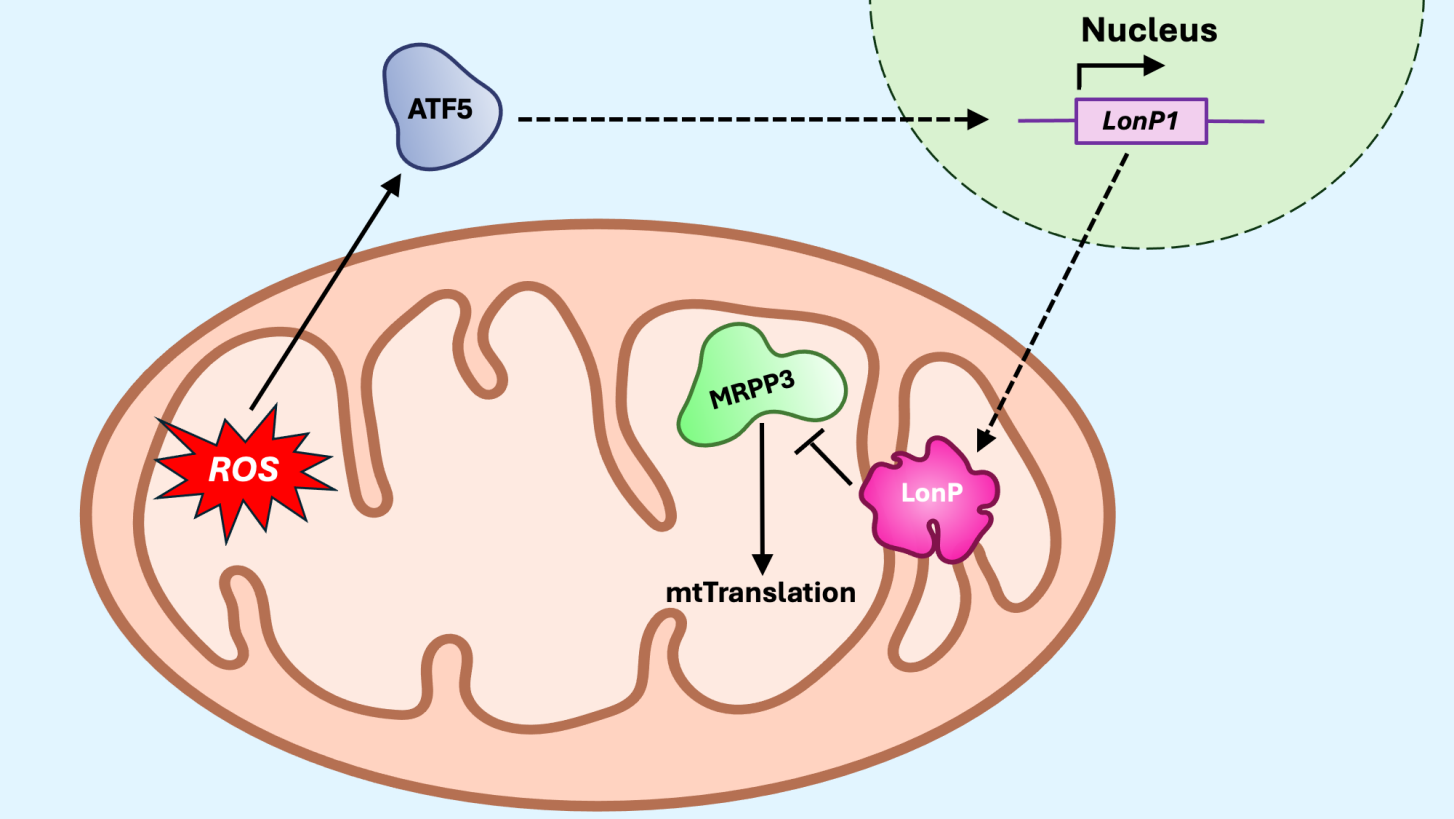
The mitoUPR translational axis. The translational axis responds to mito-nuclear protein imbalances by decreasing the synthesis of mitochondrial proteins, thereby reducing the protein-folding burden within mitochondria. Proteotoxic or oxidative stress activates transcription factor 5 (ATF) to promote transcription of Lon protease (LonP). Once in the mitochondria, LonP degrades the mitochondrial ribosomal protein MRPP3, thus reducing the protein translation within the matrix.

The translational axis of the mitoUPR helps reduce the protein synthesis in the matrix, thereby mitigating the burden on the matrix protein-folding machinery (**Figure 8**). The mitochondrial matrix protease LonP1 is critical for maintaining protein homeostasis in the mitochondrial matrix (170). Upon mitoUPR induction, LonP1 specifically degrades the mitochondrial ribonuclease P catalytic subunit 3 (MRPP3), a key nuclease involved in mitochondrial RNA processing (171). This degradation of MRPP3 leads to a reduced translation of mtDNA-encoded proteins, providing a mechanism to limit the accumulation of unfolded proteins during mitochondrial stress. Conversely, LonP activity can also promote healthy levels of translation by preventing accumulation of misfolded proteins through its protease activity implicated in the canonical axis (172). Another complementary mechanism for mitoUPR activation is the mitochondrial-nuclear protein imbalance (173), which occurs when there is a stoichiometric disparity between nuclear-encoded and mitochondrial-encoded OXPHOS subunits. While disruptions in mitochondrial protein import typically lead to reduced levels of nuclear-encoded proteins within the organelle, a mito-nuclear protein imbalance may also occur from decreased expression or translation of mitochondrial-encoded proteins, often observed when mitochondrial ribosomal proteins (MRPs) are silenced or otherwise impaired (173). In this context, microRNAs (miRNAs) have emerged as potential regulators of mitochondrial proteostasis. Evidence suggests that miRNAs may influence mitoUPR activation by modulating the expression of MRPs or other components involved in mitochondrial translation and protein folding, thus adding an additional layer of post-transcriptional regulation to the stress response machinery (174).

### mTOR-mediated regulation of mitoUPR in Tregs

The mammalian target of rapamycin (mTOR) is a master regulator of cellular metabolism and homeostasis, functioning through two distinct complexes: mTOR complex 1 (mTORC1) and mTOR complex 2 (mTORC2). Both complexes integrate nutrient availability, growth factor signals, and cellular energy status to orchestrate cell growth, differentiation, and survival (175, 176). A recent study in *C. elegans* demonstrated that mTORC1 is essential for activating the mitoUPR by sensing mitochondrial stress and increasing ATF5 transcription (177). In Treg cells, mTOR signaling plays a pivotal role in shaping metabolic programming and immune function, including mitochondrial homeostasis via the mitoUPR (15, 178, 179). Recent evidence has revealed a complex and dynamic crosstalk between mTOR signaling and the mitoUPR in Tregs, with mTORC1 and mTORC2 exerting distinct and sometimes opposing effects on this stress response pathway (15). Activation of mTORC1 enhances the expression of mitoUPR components by engaging downstream effectors such as heat shock factor 1 (HSF1) and eukaryotic translation initiation factor 4E-binding protein 1 (4EBP1) (180–182). These transcriptional and translational regulators promote the expression of mitochondrial chaperones and proteases, including CHOP, ATF4, SOD2, and ClpP, facilitating the clearance and refolding of damaged proteins. This effect is sensitive to rapamycin, a specific mTORC1 inhibitor, which reverses mTORC1-driven mitoUPR activation (181, 183). In contrast, mTORC2 contributes to mitoUPR regulation primarily by modulating the activity of FOXO transcription factors. mTORC2 inhibits FOXO proteins via AKT-mediated phosphorylation, thereby restricting their nuclear localization and transcriptional activity (176, 180). This has significant implications for Treg biology, as FOXO factors – particularly FOXO1 and FOXO3 – are essential for the expression of FoxP3, the master transcription factor of Treg cells, and for the induction of key antioxidant enzymes such as superoxide dismutase 2 (SOD2) and CAT (180, 184–186). The FOXO3 axis is further modulated by SIRT1 and SIRT3, suggesting a feedback loop in which mitochondrial stress reinforces FOXO-mediated antioxidant and mitoUPR gene expression (187). Our recent studies have shown that, while short-term inhibition of mTORC1 by rapamycin can reverse acute mitochondrial damage in Tregs, prolonged metabolic stress leads to a progressive decline in both the proliferative capacity and suppressive function of Treg cells (188). These findings illustrate the metabolic plasticity of Treg cells and highlight the potential therapeutic benefit of targeting mTOR-mitoUPR signaling to enhance Treg-mediated immunoregulation in the settings of chronic inflammation, aging, or mitochondrial dysfunction.

### Potential therapeutic targeting of the mitoUPR in Tregs

Activation of mitoUPR has been shown to extend lifespan in *C. elegans* and ameliorate damage from acute injuries such as toxin exposure, ischemia, seizure-related injuries, cardiac injury, and brain trauma (128, 189–198). In contrast, targeting the mitoUPR in chronic disease and aging is likely more complex, since activation of mitoUPR alone may not be sufficient to counteract prolonged stress conditions (192, 194, 199–205). Translation of the mitoUPR targeting strategies into clinical applications will require overcoming key challenges, including (1): the limited understanding of long-term *in vivo* effects of such approaches, whether transient or toxic, which will require further pre-clinical animal models and long-term clinical studies, and (2) current lack of efficient methods for tissue-specific drug delivery. Several studies have been exploring the potential of developing mitochondria-specific drugs for therapeutic applications. Advances in mitochondrial structural biology are continuously identifying new targets for mitochondriotropic drug delivery carriers, including delocalized lipophilic cations (DLCs), Szeto–Schiller (SS) peptides, vesicle-like aggregates such as dequalinium (DQA) or triphenylphosphonium (TPP) and the use of the mitochondrial protein import machinery for therapeutic interventions (206, 207).

To date, the direct targeting of the mitoUPR to modulate Treg cell function has not been explored in clinical settings. However, substantial pre-clinical evidence support this possibility, including the improvement of Treg function and reduction of autoimmune responses after scavenging mtROS in Tregs (93), the reduced suppressor activity in Treg cells devoid of SIRT3 (72), the higher expression of mitoUPR proteins in Treg cells compared to effector Tconv (15), and the upregulation of these proteins in response to mitochondrial redox induced stress (188).

Because of the prevalent mitochondrial reliance of Treg cells compared to effector Tconv, the modulation of the mitoUPR has emerged as a potential strategy to shift the Treg/effector Tconv balance in disease-specific contexts. Therapeutic strategies targeting the mitoUPR can exert bidirectional effects depending on the clinical objective: either prevent mitochondrial repair, leading to the accumulation of damage and selective depletion of Tregs, or support mitochondrial function and improve Treg cell survival and function (190, 198, 208). This dual potential has significant therapeutic implications. In the context of cancer, where Tregs often suppress anti-tumor immunity and contribute to immune evasion, disrupting mitochondrial integrity and inhibiting mitoUPR in Tregs may amplify anti-tumor effector responses. Conversely, in transplantation, and aging-related conditions like autoimmune or neurodegenerative disorders, where excessive effector responses drive pathology, enhancing mitoUPR to support Treg stability and function may restore immune tolerance and mitigate tissue damage. Symptoms of inflammaging may be alleviated by systemic targeting of the mitoUPR to counteract mitochondrial dysfunction and restore immune homeostasis, or by selective targeting of the mitoUPR in Treg cells to reduce inflammation.

In the context of Treg cell-based adoptive immunotherapy and the optimization of *ex vivo* expansion protocols for Treg manufacturing, monitoring mitoUPR markers may offer a valuable strategy for assessing and ensuring mitochondrial health and redox balance throughout the production process. Tracking the expression of mitoUPR components during Treg expansion may offer critical insights into the oxidative stress status of the cells and the protective mechanisms engaged to maintain mitochondrial function under *in vitro* culture conditions. Comparative analyses of mitoUPR markers across different expansion conditions – including nutrient composition, oxygen tension, and pharmacologic modulators – can support the development of standardized, clinically scalable protocols that preserve Treg identity, stability, and suppressive function. Such approaches can serve as functional quality control metrics to predict *in vivo* performance and therapeutic efficacy.

While the short-term integration of mitoUPR monitoring may improve manufacturing outcomes for clinical applications, a comprehensive understanding of Treg mitochondrial bioenergetics and stress adaptation mechanisms remains essential. Deeper characterization of mitochondrial metabolism, including OXPHOS dynamics, redox signaling, and mitoUPR regulation, will be necessary to fully exploit the therapeutic potential of Tregs, particularly in chronic inflammatory diseases, autoimmunity, and transplant tolerance. Ultimately, integrating mitochondrial profiling into Treg manufacturing workflows may not only enhance cell product consistency and potency but also pave the way for next-generation Treg-based therapies with improved durability and clinical impact.
